# *tert*-Butyl *N*-[6-(*N*,*N*-dipropyl­carbamo­yl)-1,3-benzothia­zol-2-yl]carbamate

**DOI:** 10.1107/S160053681001528X

**Published:** 2010-04-30

**Authors:** Xin Fang, Can Lei, Hai-Yang Yu, Ming-Dong Huang, Jun-Dong Wang

**Affiliations:** a College of Chemistry and Chemical Engineering, Fuzhou University, Fuzhou 350108, People’s Republic of China; b Fujian Institute of Research on the Structure of Matter, State Key Laboratory of Structural Chemistry, Chinese Academy of Sciences, Fuzhou 350002, People’s Republic of China.

## Abstract

The title compound C_19_H_27_N_3_O_3_S, crystallizes with two unique mol­ecules in the asymmetric unit. The benzene ring of each benzothia­zole unit carries a dipropyl­carbamoyl substituent in the 6-position and a *tert*-butyl carbamate unit on each thia­zole ring. In the crystal structure, inter­molecular N—H⋯N and weak C—H⋯O hydrogen bonds form centrosymmetric dimers. Additional C—H⋯O contacts construct a three-dimensional network. A very weak C—H⋯π contact is also present.

## Related literature

For benzothia­zole derivatives with anti-tumor activity, see: Brantley *et al.* (2004 ▶); Ćaleta *et al.* (2009 ▶); Mortimer *et al.* (2006 ▶) and for benzothia­zolines with anti-tuberculous properties, see: Palmer *et al.* (1971 ▶). For related benzothia­zole structures, see: Lynch  (2002 ▶); Matković-Čalogović *et al.* (2003 ▶); Lei *et al.* (2010 ▶).
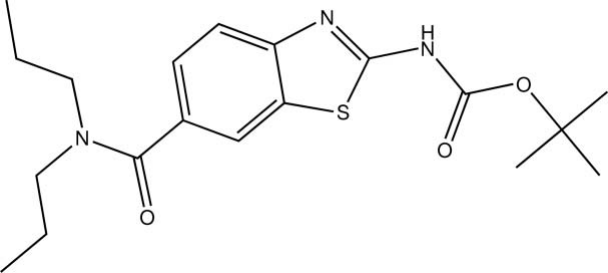


## Experimental

### 

#### Crystal data

C_19_H_27_N_3_O_3_S
*M* *_r_* = 377.50
Orthorhombic, 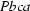

*a* = 14.068 (3) Å
*b* = 20.942 (4) Å
*c* = 26.515 (5) Å
*V* = 7812 (3) Å^3^
*Z* = 16
Mo *K*α radiation
μ = 0.19 mm^−1^
*T* = 113 K
0.45 × 0.35 × 0.23 mm

#### Data collection

Rigaku Saturn 724 CCD area-detector diffractometer
Absorption correction: numerical (*NUMABS*; Higashi, 2000 ▶) *T*_min_ = 0.993, *T*_max_ = 0.995
62834 measured reflections
8937 independent reflections
8783 reflections with *I* > 2σ(*I*)
*R*_int_ = 0.049

#### Refinement

*R*[*F* ^2^ > 2σ(*F* ^2^)] = 0.055
*wR*(*F* ^2^) = 0.114
*S* = 1.27
8937 reflections
469 parameters
H-atom parameters constrained
Δρ_max_ = 0.33 e Å^−3^
Δρ_min_ = −0.23 e Å^−3^

### 

Data collection: *CrystalClear* (Rigaku, 2007 ▶); cell refinement: *CrystalClear*; data reduction: *CrystalClear*; program(s) used to solve structure: *SHELXS97* (Sheldrick, 2008 ▶); program(s) used to refine structure: *SHELXL97* (Sheldrick, 2008 ▶); molecular graphics: *ORTEX* (McArdle, 1995 ▶); software used to prepare material for publication: *SHELXL97* and *PLATON* (Spek, 2009 ▶).

## Supplementary Material

Crystal structure: contains datablocks I, global. DOI: 10.1107/S160053681001528X/sj2775sup1.cif

Structure factors: contains datablocks I. DOI: 10.1107/S160053681001528X/sj2775Isup2.hkl

Additional supplementary materials:  crystallographic information; 3D view; checkCIF report

## Figures and Tables

**Table 1:** Hydrogen-bond geometry (Å, °) *Cg* is the centroid of the C26–C31 benzene ring.

*D*—H⋯*A*	*D*—H	H⋯*A*	*D*⋯*A*	*D*—H⋯*A*
N1—H1⋯N5	0.86	2.12	2.963 (2)	168
N4—H4⋯N2	0.86	2.16	3.006 (2)	167
C8—H8⋯O4	0.93	2.59	3.461 (2)	157
C27—H27⋯O1	0.93	2.61	3.321 (2)	134
C11—H11⋯O6^i^	0.93	2.38	3.161 (2)	141
C28—H28⋯O3^ii^	0.93	2.61	3.292 (2)	131
C37—H37*A*⋯O6^iii^	0.97	2.56	3.375 (3)	142
C16—H16*B*⋯O3^iv^	0.96	2.44	3.397 (3)	177
C20—H20*C*⋯O2^v^	0.96	2.61	3.460 (3)	147
C22—H22*A*⋯*Cg*^vi^	0.96	2.98	3.942 (3)	176

Symmetry codes: (i) 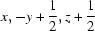; (ii) 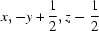; (iii) 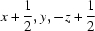; (iv) 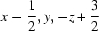; (v) 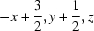; (vi) 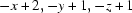.

**Fig. 1.**
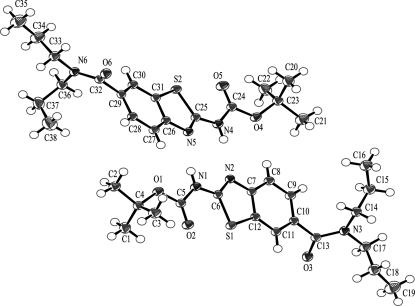
The asymmetric unit of the title compound with displacement ellipsoids for the non-hydrogen atoms drawn at the 50% probability level.

## supplementary crystallographic information

### Comment

A number of benzothiazole derivatives have anti-tuberculous (Brantley *et al.*, 2004; Mortimer *et al.*, 2006; Ćaleta *et al.*, 2009) or anti-microbial activities (Palmer *et al.*, 1971). During our development of 2-aminobenzothiazole-based Urokinase-Type Plasminogen Activator (uPA) inhibitors, the title compound was synthesized as an intermediate while its activity was not tested because it is only a fragment of our target molecule.

There are two benzothiazole molecules in one crystallographically independent unit. The benzothiazole units are similar to previously reported benzothiazole compounds (Lynch, 2002; Matković-Čalogović *et al.*, 2003), except that the two molecules are slightly distorted from a planar conformation with the angles between thiazole and benzene rings of 1.19 (7) ° for molecule 1 (C1 >> C19) and 4.01 (6) ° for molecule 2 (C20 >> C38), respectively. The dihedral angles between the carbonylamino group and the thiazole ring are 5.43 (15) °  for 1 and 18.19 (11) ° for  2, respectively. The dihedral angles between the dipropylcarbamoyl group and the benzene ring are 56.75 (16) ° for  1 and 54.0914 (1) \5 for 2, respectively.

In the crystal, molecules form pairs *via* N—H···N and C—H···O hydrogen bonds, Table 1. The dimers form a network through weak C—H···O hydrogen bonds. There is also a very weak C22—H22A···Cg contact (Cg is the centroid of the C26···C31 benzene ring). No π - π interactions are found in this structure, seemingly due to the steric hindrance of the dipropylcarbamoyl group. This is in contrast to what was found in the structure of ethyl 2-(tert-butoxycarbonylamino)-1,3-benzothiazole-6-carboxylate (Lei *et al.*, 2010).

### Experimental

A solution of ethyl 2-(tert-butoxycarbonylamino)benzo[d]thiazole-6-carboxylate (2.5 g, 7.76 mmol) was refluxed in a solution of EtOH (80 ml) and 2 N aq NaOH (50 ml) for 5 hours. Then the solution was cooled to 0^o^C and acidified with 1 N aq HCl solution. When pH < 2, white precipitate was collected, washed by water, and dried, afforded white solid of 2-(tert-butoxycarbonylamino)benzo[d]thiazole-6-carboxylic acid, N-Boc acid, (1.96 g, yield: 86%). 2-(1H-Benzotriazole-1-yl)-1,1,3,3-tetramethyluronium hexafluorophosphate, HBTU (1138 mg, 3 mmol) and N,N-Diisopropylethylamine, DIEA, (310 mg, 2.4 mmol) were added to the solution of N-Boc acid (588 mg, 2 mmol) in dry DMF (20 ml) and stired for 8 hours at room temperature, then dipropylamine (303 mg, 3 mmol) was added dropwise and the reaction continued further for 12 hours. The reaction solution was treated with water (150 ml) and then the precipitate was collected and washed with water. The filter cake was dried to yield a yellow solid and purification was achieved by column chromatography (ethyl acetate/petroleum ether 1 : 2) to yield the final product as a white solid (600 mg, yield: 79.58%). The solid was dissolved again in DMF, and filtered. After the solvent evaporated slowly at room temperature for a week, colourless rhombic crystals suitable for X-ray structure analysis were separated from the solution.

### Refinement

All H atoms bound to C and N atoms were refined as riding, with C—H distances in the range of 0.93 to 0.97 Å and N—H distances of 0.86 Å, with *U*_iso_(H) = 1.2*U*_eq_(C, N); 1.5*U*_eq_(C_methyl_).

### Crystal data

**Table:**

C_19_H_27_N_3_O_3_S	*F*(000) = 3232
*M**_r_* = 377.50	*D*_x_ = 1.284 Mg m^−^^3^
Orthorhombic, *P**b**c**a*	Mo *K*α radiation, λ = 0.71073 Å
Hall symbol: -P 2ac 2ab	Cell parameters from 28185 reflections
*a* = 14.068 (3) Å	θ = 3.0–27.5°
*b* = 20.942 (4) Å	µ = 0.19 mm^−^^1^
*c* = 26.515 (5) Å	*T* = 113 K
*V* = 7812 (3) Å^3^	Rhombic, colourless
*Z* = 16	0.45 × 0.35 × 0.23 mm

### Data collection

**Table:**

Rigaku Saturn 724 CCD area-detector diffractometer	8937 independent reflections
Radiation source: fine-focus sealed tube	8783 reflections with *I* > 2σ(*I*)
graphite	*R*_int_ = 0.049
Detector resolution: 28.5714 pixels mm^-1^	θ_max_ = 27.5°, θ_min_ = 3.0°
dtprofit.ref scans	*h* = −15→18
Absorption correction: numerical (*NUMABS*; Higashi, 2000)	*k* = −27→27
*T*_min_ = 0.993, *T*_max_ = 0.995	*l* = −30→34
62834 measured reflections	

### Refinement

**Table:**

Refinement on *F*^2^	Primary atom site location: structure-invariant direct methods
Least-squares matrix: full	Secondary atom site location: difference Fourier map
*R*[*F*^2^ > 2σ(*F*^2^)] = 0.055	Hydrogen site location: inferred from neighbouring sites
*wR*(*F*^2^) = 0.114	H-atom parameters constrained
*S* = 1.26	*w* = 1/[σ^2^(*F*_o_^2^) + (0.0264*P*)^2^ + 6.2394*P*] where *P* = (*F*_o_^2^ + 2*F*_c_^2^)/3
8937 reflections	(Δ/σ)_max_ = 0.001
469 parameters	Δρ_max_ = 0.33 e Å^−^^3^
0 restraints	Δρ_min_ = −0.23 e Å^−^^3^

### Special details

**Table:**

Geometry. All esds (except the esd in the dihedral angle between two l.s. planes) are estimated using the full covariance matrix. The cell esds are taken into account individually in the estimation of esds in distances, angles and torsion angles; correlations between esds in cell parameters are only used when they are defined by crystal symmetry. An approximate (isotropic) treatment of cell esds is used for estimating esds involving l.s. planes.
Refinement. Refinement of *F*^2^ against ALL reflections. The weighted *R*-factor wR and goodness of fit *S* are based on *F*^2^, conventional *R*-factors *R* are based on *F*, with *F* set to zero for negative *F*^2^. The threshold expression of *F*^2^ > σ(*F*^2^) is used only for calculating *R*-factors(gt) etc. and is not relevant to the choice of reflections for refinement. *R*-factors based on *F*^2^ are statistically about twice as large as those based on *F*, and *R*- factors based on ALL data will be even larger.

### Fractional atomic coordinates and isotropic or equivalent isotropic displacement parameters (Å^2^)

**Table:**

	*x*	*y*	*z*	*U*_iso_*/*U*_eq_	
S1	0.96277 (3)	0.24066 (2)	0.608747 (17)	0.02135 (10)	
S2	0.93248 (3)	0.50052 (2)	0.403321 (17)	0.02120 (10)	
O1	1.05224 (10)	0.22536 (6)	0.45079 (5)	0.0225 (3)	
O2	1.03002 (10)	0.17996 (6)	0.52814 (5)	0.0257 (3)	
O6	0.95635 (10)	0.35162 (7)	0.20407 (5)	0.0278 (3)	
O3	0.91447 (10)	0.32656 (7)	0.80962 (5)	0.0270 (3)	
O5	0.84796 (11)	0.56490 (7)	0.47992 (5)	0.0300 (3)	
O4	0.81103 (10)	0.52117 (6)	0.55639 (5)	0.0242 (3)	
N2	0.92428 (11)	0.35382 (7)	0.57104 (6)	0.0193 (3)	
N3	0.76025 (11)	0.35404 (8)	0.79978 (6)	0.0229 (3)	
N5	0.92687 (11)	0.38443 (7)	0.43988 (6)	0.0200 (3)	
N1	0.98199 (12)	0.28194 (7)	0.51118 (6)	0.0216 (3)	
H1	0.9729	0.3096	0.4877	0.026*	
N4	0.88637 (11)	0.46199 (7)	0.49931 (6)	0.0210 (3)	
H4	0.8936	0.4352	0.5236	0.025*	
N6	1.08841 (11)	0.41277 (8)	0.21075 (6)	0.0218 (3)	
C1	1.18925 (15)	0.15247 (12)	0.45972 (8)	0.0332 (5)	
H1A	1.2308	0.1888	0.4604	0.050*	
H1B	1.2216	0.1169	0.4446	0.050*	
H1C	1.1711	0.1416	0.4935	0.050*	
C2	1.12919 (15)	0.19117 (10)	0.37669 (7)	0.0279 (4)	
H2A	1.1734	0.2259	0.3796	0.042*	
H2B	1.0735	0.2053	0.3590	0.042*	
H2C	1.1583	0.1567	0.3585	0.042*	
C3	1.03136 (16)	0.11326 (10)	0.42613 (8)	0.0322 (5)	
H3A	0.9773	0.1259	0.4064	0.048*	
H3B	1.0110	0.1019	0.4595	0.048*	
H3C	1.0616	0.0772	0.4106	0.048*	
C4	1.10119 (13)	0.16828 (9)	0.42915 (7)	0.0217 (4)	
C5	1.02273 (13)	0.22401 (9)	0.49879 (7)	0.0207 (4)	
C6	0.95554 (13)	0.29683 (9)	0.55966 (7)	0.0190 (3)	
C7	0.90343 (13)	0.35638 (9)	0.62241 (7)	0.0184 (3)	
C8	0.86796 (13)	0.40970 (9)	0.64792 (7)	0.0215 (4)	
H8	0.8580	0.4480	0.6310	0.026*	
C9	0.84795 (14)	0.40442 (9)	0.69893 (7)	0.0224 (4)	
H9	0.8225	0.4393	0.7159	0.027*	
C10	0.86521 (13)	0.34754 (9)	0.72555 (7)	0.0202 (4)	
C11	0.90282 (13)	0.29492 (9)	0.70073 (7)	0.0206 (4)	
H11	0.9161	0.2574	0.7181	0.025*	
C12	0.92015 (13)	0.29959 (8)	0.64920 (7)	0.0191 (3)	
C13	0.84813 (13)	0.34180 (9)	0.78161 (7)	0.0209 (4)	
C14	0.67510 (14)	0.35806 (10)	0.76828 (7)	0.0251 (4)	
H14A	0.6296	0.3264	0.7796	0.030*	
H14B	0.6922	0.3478	0.7338	0.030*	
C15	0.62774 (16)	0.42374 (10)	0.76926 (8)	0.0308 (5)	
H15A	0.5810	0.4249	0.7961	0.037*	
H15B	0.6753	0.4560	0.7763	0.037*	
C16	0.57946 (17)	0.43906 (11)	0.71957 (8)	0.0336 (5)	
H16A	0.5506	0.4805	0.7216	0.050*	
H16B	0.5315	0.4076	0.7127	0.050*	
H16C	0.6257	0.4388	0.6930	0.050*	
C17	0.74676 (15)	0.35034 (10)	0.85472 (7)	0.0273 (4)	
H17A	0.6920	0.3759	0.8640	0.033*	
H17B	0.8020	0.3684	0.8713	0.033*	
C18	0.73213 (17)	0.28227 (11)	0.87350 (8)	0.0346 (5)	
H18A	0.7827	0.2553	0.8607	0.041*	
H18B	0.6723	0.2660	0.8607	0.041*	
C19	0.7314 (2)	0.27955 (14)	0.93096 (9)	0.0501 (7)	
H19A	0.7220	0.2362	0.9417	0.075*	
H19B	0.6808	0.3057	0.9436	0.075*	
H19C	0.7911	0.2950	0.9437	0.075*	
C20	0.66237 (15)	0.57762 (11)	0.53708 (8)	0.0319 (5)	
H20A	0.6306	0.5371	0.5368	0.048*	
H20B	0.6838	0.5877	0.5036	0.048*	
H20C	0.6191	0.6100	0.5485	0.048*	
C21	0.71580 (18)	0.55301 (12)	0.62447 (8)	0.0382 (5)	
H21A	0.6820	0.5133	0.6218	0.057*	
H21B	0.6751	0.5848	0.6391	0.057*	
H21C	0.7708	0.5473	0.6454	0.057*	
C22	0.80034 (16)	0.63702 (11)	0.57473 (10)	0.0381 (5)	
H22A	0.8531	0.6329	0.5975	0.057*	
H22B	0.7586	0.6701	0.5865	0.057*	
H22C	0.8234	0.6478	0.5417	0.057*	
C23	0.74685 (14)	0.57458 (9)	0.57230 (7)	0.0238 (4)	
C24	0.84771 (13)	0.52107 (9)	0.50973 (7)	0.0217 (4)	
C25	0.91377 (13)	0.44426 (9)	0.45142 (7)	0.0194 (3)	
C26	0.95101 (13)	0.37896 (9)	0.38902 (7)	0.0187 (3)	
C27	0.96320 (13)	0.32156 (9)	0.36315 (7)	0.0211 (4)	
H27	0.9565	0.2827	0.3798	0.025*	
C28	0.98538 (13)	0.32322 (9)	0.31227 (7)	0.0206 (4)	
H28	0.9914	0.2851	0.2945	0.025*	
C29	0.99895 (12)	0.38148 (9)	0.28707 (7)	0.0191 (3)	
C30	0.98720 (13)	0.43903 (9)	0.31258 (7)	0.0200 (4)	
H30	0.9967	0.4778	0.2962	0.024*	
C31	0.96087 (13)	0.43706 (9)	0.36330 (7)	0.0196 (4)	
C32	1.01427 (13)	0.38019 (8)	0.23074 (7)	0.0196 (4)	
C33	1.09844 (15)	0.41331 (10)	0.15547 (7)	0.0270 (4)	
H33A	1.0363	0.4072	0.1404	0.032*	
H33B	1.1382	0.3777	0.1454	0.032*	
C34	1.14122 (19)	0.47439 (11)	0.13533 (9)	0.0399 (5)	
H34A	1.2048	0.4798	0.1489	0.048*	
H34B	1.1031	0.5105	0.1461	0.048*	
C35	1.1456 (2)	0.47286 (14)	0.07794 (9)	0.0505 (7)	
H35A	1.1730	0.5119	0.0658	0.076*	
H35B	1.0826	0.4682	0.0646	0.076*	
H35C	1.1841	0.4375	0.0673	0.076*	
C36	1.17332 (14)	0.42832 (10)	0.24024 (8)	0.0268 (4)	
H36A	1.1567	0.4292	0.2757	0.032*	
H36B	1.1952	0.4706	0.2309	0.032*	
C37	1.25318 (16)	0.38125 (12)	0.23238 (9)	0.0386 (5)	
H37A	1.3078	0.3947	0.2521	0.046*	
H37B	1.2715	0.3816	0.1971	0.046*	
C38	1.2265 (2)	0.31399 (13)	0.24729 (12)	0.0516 (7)	
H38A	1.2795	0.2861	0.2415	0.077*	
H38B	1.1733	0.3001	0.2274	0.077*	
H38C	1.2096	0.3131	0.2824	0.077*	

### Atomic displacement parameters (Å^2^)

**Table:**

	*U*^11^	*U*^22^	*U*^33^	*U*^12^	*U*^13^	*U*^23^
S1	0.0280 (2)	0.0193 (2)	0.0168 (2)	0.00554 (18)	0.00337 (17)	0.00088 (16)
S2	0.0278 (2)	0.0174 (2)	0.0184 (2)	0.00189 (17)	0.00406 (17)	0.00045 (16)
O1	0.0295 (7)	0.0212 (6)	0.0169 (6)	0.0050 (5)	0.0044 (5)	−0.0015 (5)
O2	0.0333 (8)	0.0220 (7)	0.0217 (7)	0.0055 (6)	0.0063 (6)	0.0019 (5)
O6	0.0283 (7)	0.0346 (8)	0.0204 (7)	−0.0086 (6)	−0.0001 (6)	−0.0030 (6)
O3	0.0263 (7)	0.0340 (8)	0.0207 (7)	0.0040 (6)	−0.0011 (5)	0.0021 (6)
O5	0.0414 (9)	0.0227 (7)	0.0259 (7)	0.0060 (6)	0.0078 (6)	0.0032 (6)
O4	0.0314 (7)	0.0238 (7)	0.0174 (6)	0.0085 (6)	0.0028 (5)	−0.0022 (5)
N2	0.0209 (7)	0.0198 (7)	0.0171 (7)	0.0013 (6)	0.0015 (6)	−0.0005 (6)
N3	0.0233 (8)	0.0284 (8)	0.0169 (7)	0.0039 (7)	0.0020 (6)	−0.0011 (6)
N5	0.0234 (8)	0.0204 (7)	0.0161 (7)	0.0008 (6)	0.0009 (6)	0.0001 (6)
N1	0.0288 (8)	0.0199 (8)	0.0162 (7)	0.0048 (6)	0.0034 (6)	0.0014 (6)
N4	0.0266 (8)	0.0194 (7)	0.0169 (7)	0.0039 (6)	0.0019 (6)	0.0010 (6)
N6	0.0234 (8)	0.0245 (8)	0.0175 (8)	−0.0032 (6)	0.0020 (6)	−0.0011 (6)
C1	0.0271 (10)	0.0465 (13)	0.0259 (10)	0.0118 (9)	−0.0002 (8)	−0.0034 (9)
C2	0.0350 (11)	0.0284 (10)	0.0203 (9)	0.0040 (8)	0.0051 (8)	−0.0030 (8)
C3	0.0364 (12)	0.0266 (10)	0.0336 (11)	−0.0032 (9)	0.0061 (9)	−0.0082 (9)
C4	0.0237 (9)	0.0218 (9)	0.0197 (9)	0.0048 (7)	0.0040 (7)	−0.0040 (7)
C5	0.0224 (9)	0.0217 (9)	0.0181 (8)	0.0017 (7)	0.0024 (7)	−0.0021 (7)
C6	0.0192 (8)	0.0201 (8)	0.0178 (8)	0.0002 (7)	0.0008 (7)	0.0007 (7)
C7	0.0186 (8)	0.0203 (8)	0.0163 (8)	−0.0008 (7)	−0.0004 (6)	0.0003 (7)
C8	0.0249 (9)	0.0187 (8)	0.0209 (9)	0.0008 (7)	0.0006 (7)	0.0011 (7)
C9	0.0261 (9)	0.0191 (9)	0.0218 (9)	0.0020 (7)	0.0031 (7)	−0.0029 (7)
C10	0.0195 (8)	0.0224 (9)	0.0187 (9)	0.0005 (7)	0.0006 (7)	−0.0010 (7)
C11	0.0218 (9)	0.0206 (9)	0.0194 (9)	0.0027 (7)	0.0007 (7)	0.0014 (7)
C12	0.0189 (8)	0.0183 (8)	0.0199 (9)	0.0027 (7)	0.0011 (7)	0.0000 (7)
C13	0.0243 (9)	0.0191 (8)	0.0195 (9)	0.0005 (7)	0.0017 (7)	−0.0016 (7)
C14	0.0242 (9)	0.0287 (10)	0.0223 (9)	0.0013 (8)	0.0007 (7)	−0.0035 (8)
C15	0.0342 (11)	0.0309 (11)	0.0273 (10)	0.0078 (9)	−0.0028 (9)	−0.0054 (8)
C16	0.0384 (12)	0.0335 (11)	0.0290 (11)	0.0081 (9)	−0.0033 (9)	−0.0025 (9)
C17	0.0303 (10)	0.0336 (11)	0.0180 (9)	0.0060 (8)	0.0048 (8)	−0.0014 (8)
C18	0.0367 (12)	0.0394 (12)	0.0277 (11)	−0.0024 (10)	0.0070 (9)	0.0043 (9)
C19	0.0644 (18)	0.0561 (16)	0.0300 (12)	−0.0062 (14)	0.0089 (12)	0.0132 (11)
C20	0.0279 (11)	0.0354 (11)	0.0323 (11)	0.0053 (9)	−0.0047 (8)	−0.0062 (9)
C21	0.0460 (13)	0.0434 (13)	0.0252 (10)	0.0162 (11)	0.0070 (9)	−0.0043 (9)
C22	0.0303 (11)	0.0322 (11)	0.0518 (14)	−0.0021 (9)	0.0020 (10)	−0.0180 (10)
C23	0.0244 (9)	0.0229 (9)	0.0240 (9)	0.0062 (7)	0.0009 (7)	−0.0059 (7)
C24	0.0227 (9)	0.0225 (9)	0.0199 (9)	0.0018 (7)	0.0001 (7)	−0.0018 (7)
C25	0.0197 (8)	0.0205 (8)	0.0180 (8)	0.0011 (7)	0.0008 (7)	0.0000 (7)
C26	0.0194 (8)	0.0210 (8)	0.0157 (8)	0.0002 (7)	−0.0003 (6)	0.0010 (7)
C27	0.0255 (9)	0.0183 (8)	0.0194 (9)	−0.0007 (7)	−0.0002 (7)	0.0019 (7)
C28	0.0219 (9)	0.0195 (9)	0.0203 (9)	0.0002 (7)	−0.0017 (7)	−0.0017 (7)
C29	0.0174 (8)	0.0224 (9)	0.0174 (8)	0.0007 (7)	0.0002 (6)	−0.0005 (7)
C30	0.0231 (9)	0.0187 (8)	0.0183 (8)	0.0010 (7)	0.0025 (7)	0.0030 (7)
C31	0.0197 (8)	0.0191 (8)	0.0200 (9)	0.0019 (7)	0.0001 (7)	−0.0005 (7)
C32	0.0210 (9)	0.0176 (8)	0.0203 (9)	0.0013 (7)	0.0007 (7)	−0.0005 (7)
C33	0.0296 (10)	0.0330 (11)	0.0184 (9)	−0.0044 (8)	0.0027 (7)	0.0008 (8)
C34	0.0531 (15)	0.0351 (12)	0.0314 (12)	−0.0038 (11)	0.0093 (10)	0.0054 (10)
C35	0.0652 (18)	0.0556 (16)	0.0306 (12)	−0.0087 (14)	0.0093 (12)	0.0127 (11)
C36	0.0239 (10)	0.0316 (10)	0.0250 (10)	−0.0064 (8)	0.0016 (8)	−0.0057 (8)
C37	0.0227 (10)	0.0552 (15)	0.0378 (12)	0.0055 (10)	−0.0008 (9)	−0.0078 (11)
C38	0.0442 (15)	0.0456 (14)	0.0649 (18)	0.0216 (12)	−0.0073 (13)	−0.0014 (13)

### Geometric parameters (Å, °)

**Table:**

S1—C12	1.7416 (18)	C15—H15A	0.9700
S1—C6	1.7574 (18)	C15—H15B	0.9700
S2—C31	1.7469 (18)	C16—H16A	0.9600
S2—C25	1.7563 (19)	C16—H16B	0.9600
O1—C5	1.339 (2)	C16—H16C	0.9600
O1—C4	1.494 (2)	C17—C18	1.524 (3)
O2—C5	1.211 (2)	C17—H17A	0.9700
O6—C32	1.234 (2)	C17—H17B	0.9700
O3—C13	1.235 (2)	C18—C19	1.525 (3)
O5—C24	1.211 (2)	C18—H18A	0.9700
O4—C24	1.341 (2)	C18—H18B	0.9700
O4—C23	1.498 (2)	C19—H19A	0.9600
N2—C6	1.307 (2)	C19—H19B	0.9600
N2—C7	1.394 (2)	C19—H19C	0.9600
N3—C13	1.351 (2)	C20—C23	1.513 (3)
N3—C14	1.463 (2)	C20—H20A	0.9600
N3—C17	1.471 (2)	C20—H20B	0.9600
N5—C25	1.303 (2)	C20—H20C	0.9600
N5—C26	1.395 (2)	C21—C23	1.519 (3)
N1—C6	1.374 (2)	C21—H21A	0.9600
N1—C5	1.381 (2)	C21—H21B	0.9600
N1—H1	0.8600	C21—H21C	0.9600
N4—C25	1.378 (2)	C22—C23	1.510 (3)
N4—C24	1.379 (2)	C22—H22A	0.9600
N4—H4	0.8600	C22—H22B	0.9600
N6—C32	1.354 (2)	C22—H22C	0.9600
N6—C36	1.464 (2)	C26—C27	1.395 (2)
N6—C33	1.473 (2)	C26—C31	1.401 (2)
C1—C4	1.517 (3)	C27—C28	1.385 (3)
C1—H1A	0.9600	C27—H27	0.9300
C1—H1B	0.9600	C28—C29	1.404 (3)
C1—H1C	0.9600	C28—H28	0.9300
C2—C4	1.523 (3)	C29—C30	1.392 (3)
C2—H2A	0.9600	C29—C32	1.509 (2)
C2—H2B	0.9600	C30—C31	1.396 (2)
C2—H2C	0.9600	C30—H30	0.9300
C3—C4	1.516 (3)	C33—C34	1.511 (3)
C3—H3A	0.9600	C33—H33A	0.9700
C3—H3B	0.9600	C33—H33B	0.9700
C3—H3C	0.9600	C34—C35	1.523 (3)
C7—C8	1.398 (2)	C34—H34A	0.9700
C7—C12	1.405 (2)	C34—H34B	0.9700
C8—C9	1.386 (3)	C35—H35A	0.9600
C8—H8	0.9300	C35—H35B	0.9600
C9—C10	1.406 (3)	C35—H35C	0.9600
C9—H9	0.9300	C36—C37	1.509 (3)
C10—C11	1.388 (3)	C36—H36A	0.9700
C10—C13	1.511 (2)	C36—H36B	0.9700
C11—C12	1.391 (2)	C37—C38	1.511 (4)
C11—H11	0.9300	C37—H37A	0.9700
C14—C15	1.528 (3)	C37—H37B	0.9700
C14—H14A	0.9700	C38—H38A	0.9600
C14—H14B	0.9700	C38—H38B	0.9600
C15—C16	1.517 (3)	C38—H38C	0.9600
			
C12—S1—C6	87.81 (9)	C17—C18—H18B	109.4
C31—S2—C25	87.99 (9)	C19—C18—H18B	109.4
C5—O1—C4	119.41 (14)	H18A—C18—H18B	108.0
C24—O4—C23	119.54 (14)	C18—C19—H19A	109.5
C6—N2—C7	109.36 (15)	C18—C19—H19B	109.5
C13—N3—C14	123.81 (16)	H19A—C19—H19B	109.5
C13—N3—C17	117.46 (16)	C18—C19—H19C	109.5
C14—N3—C17	117.57 (16)	H19A—C19—H19C	109.5
C25—N5—C26	109.89 (15)	H19B—C19—H19C	109.5
C6—N1—C5	122.29 (15)	C23—C20—H20A	109.5
C6—N1—H1	118.9	C23—C20—H20B	109.5
C5—N1—H1	118.9	H20A—C20—H20B	109.5
C25—N4—C24	122.45 (16)	C23—C20—H20C	109.5
C25—N4—H4	118.8	H20A—C20—H20C	109.5
C24—N4—H4	118.8	H20B—C20—H20C	109.5
C32—N6—C36	122.09 (16)	C23—C21—H21A	109.5
C32—N6—C33	117.85 (16)	C23—C21—H21B	109.5
C36—N6—C33	116.85 (16)	H21A—C21—H21B	109.5
C4—C1—H1A	109.5	C23—C21—H21C	109.5
C4—C1—H1B	109.5	H21A—C21—H21C	109.5
H1A—C1—H1B	109.5	H21B—C21—H21C	109.5
C4—C1—H1C	109.5	C23—C22—H22A	109.5
H1A—C1—H1C	109.5	C23—C22—H22B	109.5
H1B—C1—H1C	109.5	H22A—C22—H22B	109.5
C4—C2—H2A	109.5	C23—C22—H22C	109.5
C4—C2—H2B	109.5	H22A—C22—H22C	109.5
H2A—C2—H2B	109.5	H22B—C22—H22C	109.5
C4—C2—H2C	109.5	O4—C23—C22	110.99 (16)
H2A—C2—H2C	109.5	O4—C23—C20	109.34 (15)
H2B—C2—H2C	109.5	C22—C23—C20	112.42 (18)
C4—C3—H3A	109.5	O4—C23—C21	101.98 (15)
C4—C3—H3B	109.5	C22—C23—C21	111.22 (18)
H3A—C3—H3B	109.5	C20—C23—C21	110.41 (18)
C4—C3—H3C	109.5	O5—C24—O4	127.02 (17)
H3A—C3—H3C	109.5	O5—C24—N4	123.22 (17)
H3B—C3—H3C	109.5	O4—C24—N4	109.76 (16)
O1—C4—C3	109.24 (15)	N5—C25—N4	121.01 (16)
O1—C4—C1	110.23 (15)	N5—C25—S2	116.96 (14)
C3—C4—C1	113.05 (18)	N4—C25—S2	122.03 (14)
O1—C4—C2	102.60 (14)	C27—C26—N5	125.16 (16)
C3—C4—C2	111.01 (17)	C27—C26—C31	119.80 (16)
C1—C4—C2	110.21 (17)	N5—C26—C31	115.03 (16)
O2—C5—O1	126.91 (17)	C28—C27—C26	119.02 (17)
O2—C5—N1	123.45 (17)	C28—C27—H27	120.5
O1—C5—N1	109.64 (15)	C26—C27—H27	120.5
N2—C6—N1	120.96 (16)	C27—C28—C29	121.05 (17)
N2—C6—S1	117.36 (14)	C27—C28—H28	119.5
N1—C6—S1	121.68 (14)	C29—C28—H28	119.5
N2—C7—C8	125.35 (16)	C30—C29—C28	120.34 (16)
N2—C7—C12	115.22 (16)	C30—C29—C32	120.88 (16)
C8—C7—C12	119.44 (16)	C28—C29—C32	118.35 (16)
C9—C8—C7	118.75 (17)	C29—C30—C31	118.30 (17)
C9—C8—H8	120.6	C29—C30—H30	120.8
C7—C8—H8	120.6	C31—C30—H30	120.8
C8—C9—C10	121.50 (17)	C30—C31—C26	121.38 (17)
C8—C9—H9	119.2	C30—C31—S2	128.57 (14)
C10—C9—H9	119.2	C26—C31—S2	110.02 (13)
C11—C10—C9	120.03 (17)	O6—C32—N6	121.92 (17)
C11—C10—C13	117.64 (16)	O6—C32—C29	118.79 (16)
C9—C10—C13	122.28 (16)	N6—C32—C29	119.22 (16)
C10—C11—C12	118.46 (17)	N6—C33—C34	113.35 (17)
C10—C11—H11	120.8	N6—C33—H33A	108.9
C12—C11—H11	120.8	C34—C33—H33A	108.9
C11—C12—C7	121.77 (16)	N6—C33—H33B	108.9
C11—C12—S1	127.97 (14)	C34—C33—H33B	108.9
C7—C12—S1	110.25 (13)	H33A—C33—H33B	107.7
O3—C13—N3	121.74 (17)	C33—C34—C35	110.6 (2)
O3—C13—C10	119.49 (17)	C33—C34—H34A	109.5
N3—C13—C10	118.77 (16)	C35—C34—H34A	109.5
N3—C14—C15	113.52 (16)	C33—C34—H34B	109.5
N3—C14—H14A	108.9	C35—C34—H34B	109.5
C15—C14—H14A	108.9	H34A—C34—H34B	108.1
N3—C14—H14B	108.9	C34—C35—H35A	109.5
C15—C14—H14B	108.9	C34—C35—H35B	109.5
H14A—C14—H14B	107.7	H35A—C35—H35B	109.5
C16—C15—C14	111.76 (17)	C34—C35—H35C	109.5
C16—C15—H15A	109.3	H35A—C35—H35C	109.5
C14—C15—H15A	109.3	H35B—C35—H35C	109.5
C16—C15—H15B	109.3	N6—C36—C37	112.85 (17)
C14—C15—H15B	109.3	N6—C36—H36A	109.0
H15A—C15—H15B	107.9	C37—C36—H36A	109.0
C15—C16—H16A	109.5	N6—C36—H36B	109.0
C15—C16—H16B	109.5	C37—C36—H36B	109.0
H16A—C16—H16B	109.5	H36A—C36—H36B	107.8
C15—C16—H16C	109.5	C36—C37—C38	112.81 (19)
H16A—C16—H16C	109.5	C36—C37—H37A	109.0
H16B—C16—H16C	109.5	C38—C37—H37A	109.0
N3—C17—C18	112.97 (17)	C36—C37—H37B	109.0
N3—C17—H17A	109.0	C38—C37—H37B	109.0
C18—C17—H17A	109.0	H37A—C37—H37B	107.8
N3—C17—H17B	109.0	C37—C38—H38A	109.5
C18—C17—H17B	109.0	C37—C38—H38B	109.5
H17A—C17—H17B	107.8	H38A—C38—H38B	109.5
C17—C18—C19	111.2 (2)	C37—C38—H38C	109.5
C17—C18—H18A	109.4	H38A—C38—H38C	109.5
C19—C18—H18A	109.4	H38B—C38—H38C	109.5

### Hydrogen-bond geometry (Å, °)

**Table:**

Cg is the centroid of the C26–C31 benzene ring.

**Table:**

*D*—H···*A*	*D*—H	H···*A*	*D*···*A*	*D*—H···*A*
N1—H1···N5	0.86	2.12	2.963 (2)	168
N4—H4···N2	0.86	2.16	3.006 (2)	167
C8—H8···O4	0.93	2.59	3.461 (2)	157
C27—H27···O1	0.93	2.61	3.321 (2)	134
C11—H11···O6^i^	0.93	2.38	3.161 (2)	141
C28—H28···O3^ii^	0.93	2.61	3.292 (2)	131
C37—H37A···O6^iii^	0.97	2.56	3.375 (3)	142
C16—H16B···O3^iv^	0.96	2.44	3.397 (3)	177
C20—H20C···O2^v^	0.96	2.61	3.460 (3)	147
C22—H22A···Cg^vi^	0.96	2.98	3.942 (3)	176

Symmetry codes: (i) *x*, −*y*+1/2, *z*+1/2; (ii) *x*, −*y*+1/2, *z*−1/2; (iii) *x*+1/2, *y*, −*z*+1/2; (iv) *x*−1/2, *y*, −*z*+3/2; (v) −*x*+3/2, *y*+1/2, *z*; (vi) −*x*+2, −*y*+1, −*z*+1.
